# Maternal and early life nutrition and physical activity: setting the research and intervention agenda for addressing the double burden of malnutrition in South African children

**DOI:** 10.1080/16549716.2017.1301085

**Published:** 2017-05-19

**Authors:** A. Prioreschi, S. Wrottesley, C. E. Draper, S. A. Tomaz, C. J. Cook, E. D. Watson, M. N. M. Van Poppel, R. Said-Mohamed, S. A. Norris, E. V. Lambert, L. K. Micklesfield

**Affiliations:** ^a^ MRC/WITS Developmental Pathways for Health Research Unit, Department of Paediatrics, School of Clinical Medicine, Faculty of Health Sciences, University of the Witwatersrand, Johannesburg, South Africa; ^b^ Division of Exercise Science and Sports Medicine, Department of Human Biology, Faculty of Health Sciences, University of Cape Town, Cape Town, South Africa; ^c^ Centre for Exercise Science and Sports Medicine, School of Therapeutic Sciences, Faculty of Health Sciences, University of the Witwatersrand, Johannesburg, South Africa; ^d^ Department of Public and Occupational Health, EMGO Institute for Health and Care Research, VU University Medical Center, Amsterdam, The Netherlands; ^e^ Institute of Sport Science, University of Graz, Graz, Austria

**Keywords:** Public health, developing countries, Africa, pregnancy, infancy

## Abstract

Early life is important for later health outcomes, yet there are few studies which adequately address all of the potential early life insults that may affect later life health and growth trajectories. This is particularly evident in low- to middle-income countries such as South Africa, where women of childbearing age are particularly vulnerable to high levels of physical inactivity, malnutrition, and obesity. Pregnancy may therefore be an opportune time to change behaviours and improve maternal and offspring health outcomes, and decrease the inter-generational transfer of risk. We show clear evidence that physical activity and nutrition are important target areas for intervention during pregnancy and in the early years of life, yet that current literature in Africa, and specifically South Africa, is limited. We have outlined the available literature concerning the impact of maternal and early life nutrition and physical activity on the health status of South African children, and have provided some recommendations for future research and policy.

## Background

Early life (including the gestational period) has been shown to be important for later health outcomes. There is growing evidence that certain chronic diseases have their origin in early life [1], as a result of either foetal and epi-genetic programming, maternal nutritional insults, and/or tracking of certain behaviours such as physical activity and dietary intake. These serial insults of early life under- or over-nutrition, followed by childhood physical inactivity and unhealthy eating, have been described as the first and second ‘hits’ in terms of early life programming of chronic non-communicable diseases (NCDs) [2]. In many developing African countries, rapid urbanisation and ongoing behavioural transitions have resulted in a dual burden of over- and undernutrition [3,4]. South Africa is a particularly unique setting where the prevalence of overweight and obesity is starting to predominate, while undernutrition is becoming far less frequent, and physical inactivity is rising [5–7]. These risk factors coexist with a high prevalence of NCDs and HIV, as well as the persistence of food insecurity. Women of childbearing age in South Africa are thus particularly vulnerable to high levels of physical inactivity, malnutrition, and obesity [8], and so pregnancy may be an opportune time to change these behaviours in order to improve maternal and offspring health outcomes, and decrease the inter-generational transfer of risk.

There are few studies which adequately address all of these potential sources of influence on the growth trajectory of children and later adult health and health risk status, particularly in lower- and middle-income country (LMIC) settings. Here we address the limited available evidence concerning these early life factors in South Africa and highlight the need for systematic and focused research, to intervene not only for early life gains for childhood development and social capital, but for later life benefits by attenuating the risk for NCDs. This research agenda is particularly timely in light of the United Nations’ (UN’s) Sustainable Development Goals (SDGs) for 2030 [9].

## Maternal nutrition and physical activity during pregnancy

The International Federation of Gynecology and Obstetrics (FIGO) in their recent supplement [10] have identified the importance of maternal nutrition and physical activity during pregnancy for ensuring appropriate gestational weight gain (GWG). Maternal nutritional status is a strong predictor of foetal growth and development, with maternal obesity, adiposity, and excessive GWG in particular being associated not only with increased risk of gestational diabetes mellitus (GDM) and pre-eclampsia during pregnancy, but also with adverse birth outcomes such as pre-term birth, still birth, and macrosomia (birth weight > 4 kg) [11,12]. Sub-optimal growth and development, as well as exposure to pregnancy-associated metabolic risk factors, may increase susceptibility to NCDs in later life for both mother and infant [13,14]. Pilot data from the Soweto First 1000 Days Study shows that, within an urban-poor South African context, women exhibit a high clustering of disease during pregnancy, including 67% overweight and obesity, 14% GDM, and 31% anaemia (Norris et al., unpublished). Therefore, although intervening during the pre-conception period is key, intervention during pregnancy is still an important area of focus, and may drive critical improvements in maternal and child health, as well as metabolic risk in the longer term.

However, intervention studies which examine the role of nutrition during pregnancy in improving maternal and infant outcomes in transitioning communities are scarce. Although African settings exhibit features typical of the epidemiological transition, such as higher prevalences of maternal overweight and obesity, combined with poor diet quality and multiple micronutrient deficiencies, the majority of trials focus on macro- and micronutrient supplementation in chronically malnourished women [15]. Only one South African study conducted in the Vaal Triangle, which is a semi-industrialised, low-income region, showed that pregnant women were predominantly overweight/obese and consumed diets which were high in energy and refined sugars, low in protein and micronutrients, and lacked diversity [16]. FIGO has highlighted the importance of a diverse diet during pregnancy, particularly in LMICs where diets are often based on staples and are unvarying, even while calorie intake is high [10]. There has been a lack of focus on the effects of this ‘hidden hunger’ on maternal and infant outcomes in both the short and long term, as well as on effective means of intervention in South Africa, and future work should thus examine dietary intake and micronutrient marker patterns and associations with maternal and infant outcomes – as well as the associated risk profiles that may track into adulthood. Further, interventions which aim to improve dietary diversity and quality in South African pregnant women, including appropriate energy intakes with micronutrient-rich foods and limited refined sugar (< 10%), salt (< 5%), and convenience foods, should be explored. Specifically, FIGO recommends intake of > 15 g of at least five of the following: starchy staples, beans and peas, nuts and seeds, dairy, meat, poultry and fish, eggs, vitamin A-rich vegetables and fruits, and other vegetables and fruits [10].

A large body of research has shown that physical activity during pregnancy provides many maternal health benefits, such as management of GWG, and reduction in the risk of GDM as well as pre-eclampsia [17,18]. The exact effect of maternal physical activity on foetal growth and development remains controversial [1,19,20], yet it is essential that pregnant women are physically active enough to maintain a healthy bodyweight and prevent increased GWG, particularly when pre-pregnancy body mass index (BMI) are high [10]. Much of the available intervention literature is from high-income countries (HICs), with only a small proportion originating from LMICs [17], and none coming from Africa. A recently conducted systematic review found only five studies that had measured physical activity during pregnancy in Africa; moreover, none of these were intervention studies [21].

The South African Sports Medicine Association recommends participating in 150 minutes of moderate-intensity physical activity per week during pregnancy [22]; however, African pregnant women may be at risk for not meeting these guidelines. Hjorth et al. have shown that pregnant women from Ethiopia spend the majority of their time in sedentary behaviours, and appear to do less physical activity than non-pregnant African women, and less than pregnant women from HICs [23]. In addition, it is well-known that the patterns and types of physical activity performed in LMICs are unique. Many African women participate in occupational, domestic, or farming activities, and may continue these activities during their pregnancies [24,25]. Indeed, recent South African data has shown that much of the physical activity performed during pregnancy comes from walking for transport, with little coming from recreational physical activity [26]; however, little is known about the effect of these patterns of activity on maternal and birth outcomes. Recent data has shown that only 51% of pregnant women from a Sowetan cohort meet the physical activity recommendations in the second trimester [26], when taking all types of activity into account. These findings are similar to national findings from non-pregnant South African females [7]; however, physical activity levels have been shown to decline significantly throughout the course of the gestational period [26], putting women in the later stages of pregnancy at even higher risk of insufficient physical activity. Two South African qualitative studies have found that a lack of a supportive environment, inadequate knowledge and education, as well as conflicting cultural beliefs are all possible reasons for the declining levels of physical activity during pregnancy [27,28].

To the best of our knowledge, all the maternal physical activity research within the African context has been observational, and well-designed intervention studies are now needed in this setting. Preliminary South African data has shown that higher total physical activity during pregnancy may assist with managing GWG, which is important in a population with a high prevalence of obesity in early pregnancy, whilst posing no risk to the growing foetus [26]. Over and above well-designed intervention studies, it is important to address potential barriers to physical activity participation during pregnancy (for example, creating supportive environments and addressing perceived risks to personal safety). Intervention studies should strive to incorporate lessons learnt from HIC research studies that have often suffered from poor compliance, especially towards the end of pregnancy [29]. It is important to note that, while nutrition and physical activity interventions during pregnancy are necessary for the improvement of pregnancy outcomes, even well-designed interventions have not been able to sufficiently limit GWG in overweight/obese pregnant women [30], and intervention during the preconception period should be the focus of future public health strategies.

## Nutrition and physical activity during infancy and childhood

In the first five years of life, the double burden of under- and over-nutrition in South Africa is evident [31,32]. In 2013, 22.9% of 2–5-year-old children were reported to be overweight or obese, and the prevalence of stunting was 21.5%. However in children 0–3 years, the prevalences of stunting were as high as 26.9% for boys and 25.9% for girls [33]. An earlier study showed that the co-prevalence of stunting and overweight in 3-year-old children was 19% [32]. Research in LMICs suggests that stunted children have impaired lipid metabolism favouring the accumulation of adipose tissue, and that in the context of high energy intake and low energy expenditure, undernourished children could be at risk for developing overweight/obesity [34–36].

In the first two years of life specifically, international data has shown that breastfeeding has beneficial effects on maternal and child health [37]. Global policies prescribe exclusive breastfeeding for the first six months of life and the continuation of breastfeeding for two years or beyond, and that complementary feeding should introduce dietary variation and nutrient-rich foods [10]. South African data is limited, yet shows that most mothers do initiate breastfeeding immediately after delivery, but that this does not remain exclusive for six months [38]. Breastfeeding policies have been complicated by the high prevalence of HIV in South Africa, and concerns regarding mother to child transmission that resulted in rapid policy changes and confusion as to which guidelines to follow. Research from South Africa has informed current policies for HIV-positive women, which recommend exclusive breastfeeding for the first six months (while taking antiretroviral therapy), and for breastfeeding to continue up until one year [38]. Implementation of these policy recommendations should now be the focus of nutrition interventions in South Africa in the first two years of life. There are a number of relevant policies and guidelines that inform interventions (e.g. Roadmap for Nutrition in South Africa: 2013–2017; Infant and Young Child Feeding Policy) [39,40], aiming to assist with the reduction of both all forms of undernutrition, as well as overweight and obesity. However, the extent of their implementation, and evaluation of specific interventions remain areas for improvement.

In early childhood studies (mostly from HICs), higher physical activity levels have been associated with favourable measures of body composition [41,42] and other health outcomes relating to decreased NCD risk [1,43], as well as with greater gross motor competence [1]. Sedentary behaviour has also been associated with obesity in childhood [41,42]. Studies assessing physical activity levels in the first two years of life are limited; however, there is evidence to suggest that physical activity and sedentary behaviours track through childhood [44,45], and into adulthood [46,47], making it crucial that healthy behaviours are established early in life. Although it has typically been assumed that young children are naturally active enough to be protected against overweight and obesity, evidence regarding preschool children’s physical activity levels challenges this assumption [48], and the sparsity of data in younger children makes evidence-based investigation necessary.

In South Africa, to our knowledge no studies have measured physical activity in the first two years of life. Only one published study has assessed physical activity in preschools, reporting a high proportion of time spent in sedentary behaviours and indoors while at preschool [49]. This clear gap in the current literature highlights the need for further research measuring physical activity levels and patterns in the first two years of life and in the preschool years (±3–5 years), as well as effects on childhood and later health in South African children. Furthermore, maternal beliefs regarding physical activity in children have not been investigated. Until we have a clear understanding of normal activity behaviours in this age group and associations with health outcomes, we are unable to intervene appropriately. The recent 2016 Healthy Active Kids South Africa (HAKSA) report card has shown that physical activity levels in South African children are of concern, with only half of all children assessed meeting recommendations [50]. Furthermore, sedentary behaviour is high, and there is insufficient evidence on opportunities for active play. This research was only conducted in older children, and the HAKSA report has highlighted the need to conduct further research examining physical activity levels and sedentary behaviour in early childhood.

In South Africa, there are no documented obesity prevention (or management) interventions in early childhood that integrate nutrition, physical activity and sedentary behaviour, and maternal education that have been rigorously developed and evaluated. In this regard, national surveys are helpful for tracking trends in children’s nutritional status and associated factors, including physical activity and sedentary behaviour. Some formative research has been conducted to assess physical activity and gross motor skills in preschool children in South Africa from urban (high- and low-income) and rural settings. These findings are currently being prepared for publication, and are informing the development of integrated nutrition and physical activity intervention strategies, involving preschool teachers, parents, and caregivers.

## Conclusion and recommendations

In conclusion, there is clear evidence that physical activity and nutrition are important target areas for intervention during pregnancy and in the early years of life, yet current literature in South Africa is limited and places these periods of life at different developmental stages of research, intervention, and implementation. Based on the currently available literature we recommend the following:

1. Future research should examine maternal dietary patterns and the effects on maternal weight gain and infant outcomes (such as birth weight and pre-term birth).
2. Research needs to be conducted into levels of physical activity and sedentary behaviour in infants and young children, and associations with growth, development, and health.
3. Interventions should focus on creating supportive environments for infant and child play, which should include maternal education as well as preschool physical education curricula development and implementation on a national level.
4. Pregnant women should be advised to maintain 150 min of moderate intensity physical activity per week, or to increase physical activity levels to meet this target. Furthermore, interventions should focus on creating supportive environments for women to increase or maintain their physical activity levels during pregnancy.
5. Policies should prescribe maintenance or introduction of a healthy and diverse diet during pregnancy, with a focus on maintaining calorie intake at a level that will not increase GWG above the globally recommended levels, especially in women who are already overweight or obese.
6. Focus should be placed on implementation of breastfeeding and complementary feeding policies, and nutrition policies during early childhood.
7. Interventions and policies should ultimately focus on the preconception period for optimal impact.

We have thus outlined the available literature concerning the effects of maternal and early life nutrition and physical activity on the health status of South African children and have provided some recommendations for future research and policy. This provides a ‘road-map’ for research, intervention, and a research translation agenda in South Africa which could be extended to effectively address the double burden of malnutrition in young African children, and to be able to intervene effectively.

## Paper context

The importance of the early life period (pregnancy and early childhood) in setting up future health trajectories has been established. In South Africa, information on maternal and infant nutrition and physical activity is limited, and interventions have not yet been conducted. The intergenerational transfer of risk in this vulnerable population is thus of concern. This paper therefore examines current evidence, and recommends future research directions and policy pertaining to pregnancy and early childhood, from a South African context.
